# Sawfish: improving long-read structural variant discovery and genotyping with local haplotype modeling

**DOI:** 10.1093/bioinformatics/btaf136

**Published:** 2025-04-09

**Authors:** Christopher T Saunders, James M Holt, Daniel N Baker, Juniper A Lake, Jonathan R Belyeu, Zev Kronenberg, William J Rowell, Michael A Eberle

**Affiliations:** Computational Biology, PacBio, Menlo Park, CA 94025, United States; Computational Biology, PacBio, Menlo Park, CA 94025, United States; Computational Biology, PacBio, Menlo Park, CA 94025, United States; Computational Biology, PacBio, Menlo Park, CA 94025, United States; Computational Biology, PacBio, Menlo Park, CA 94025, United States; Computational Biology, PacBio, Menlo Park, CA 94025, United States; Computational Biology, PacBio, Menlo Park, CA 94025, United States; Computational Biology, PacBio, Menlo Park, CA 94025, United States

## Abstract

**Motivation:**

Structural variants (SVs) play an important role in evolutionary and functional genomics but are challenging to characterize. High-accuracy, long-read sequencing can substantially improve SV characterization when coupled with effective calling methods. While state-of-the-art long-read SV callers are highly accurate, further improvements are achievable by systematically modeling local haplotypes during SV discovery and genotyping.

**Results:**

We describe sawfish, an SV caller for mapped high-quality long reads incorporating systematic SV haplotype modeling to improve accuracy and resolution. Assessment against the draft Genome in a Bottle (GIAB) SV benchmark from the T2T-HG002-Q100 diploid assembly shows that sawfish has the highest accuracy among state-of-the-art long-read SV callers across every tested SV size group. Additionally, sawfish maintains the highest accuracy at every tested depth level from 10- to 32-fold coverage, such that other callers required at least 30-fold coverage to match sawfish accuracy at 15-fold coverage. Sawfish also shows the highest accuracy in the GIAB challenging medically relevant genes benchmark, demonstrating improvements in both comprehensive and medically relevant contexts.

When joint-genotyping seven samples from CEPH-1463, sawfish has over 9000 more pedigree-concordant calls than other state-of-the-art SV callers, with the highest proportion of concordant SVs (81%). Sawfish’s quality model enables selection for an even higher proportion of concordant SVs (88%), while still calling nearly 5000 more pedigree-concordant SVs than other callers. These results demonstrate that sawfish improves on the state-of-the-art for long-read SV calling accuracy across both individual and joint-sample analyses.

**Availability and implementation:**

Sawfish source code, pre-compiled Linux binaries, and documentation are released on GitHub: https://github.com/PacificBiosciences/sawfish.

## 1 Introduction

Structural variants (SVs) are medium- to large-scale rearrangements of the genome, typically characterized as events at least 50 bases in size. Though there are relatively few SVs in the human genome compared to the number of single-nucleotide variants and small indels, collectively these SVs impact more bases than small variants and play a significant role in human evolution and disease (Sudmant *et al.* 2015). SVs are often mediated by repetitive sequence that can be difficult to study with short-read sequencing technologies (Chaisson *et al.* 2019, Logsdon *et al.* 2020, Zhao *et al.* 2021), so our understanding of these variants has been limited compared to smaller variants. Recent advances in highly accurate long-read sequencing together with updated SV detection methods have greatly improved the quality of SVs routinely characterized from human samples (Wenger *et al.* 2019, Logsdon *et al.* 2020), but continued improvements to SV characterization methods are critical to realizing the full potential of long-read genome sequencing.

We describe a new method, sawfish, which assembles SV haplotypes and models variants at the haplotype level in all downstream sample merging and genotyping steps. We show that such an approach can substantially improve SV accuracy on the latest high-resolution assembly-based SV benchmarks. Compared to other state-of-the-art long-read SV callers on these benchmarks, sawfish provides the most accurate SV calls in aggregate as well as across SV size ranges. We also show that sawfish provides the most accurate SV calls at every tested sequencing depth from 10- to 32-fold coverage, allowing sawfish with 15-fold sequencing coverage to match the accuracy of other callers at 30-fold coverage. We additionally show that sawfish’s approach leads to higher genotype concordance on a large pedigree, where sawfish not only produces thousands more concordant SVs than other callers, but also a greater proportion of concordant SVs, indicative of a more accurate genotyping model. In aggregate, our results show that sawfish’s methods comprehensively improve the accuracy of long-read SV calling in both single- and joint-sample analysis scenarios.

## 2 Materials and methods

Sawfish is capable of calling and genotyping deletions, insertions, duplications, translocations, and inversions from mapped high-accuracy long reads. The method is designed to discover breakpoint evidence from each sample, then merge and genotype variant calls across samples in a subsequent joint-genotyping step, using a process that emphasizes representation of each SV’s local haplotype sequence to improve accuracy. The primary sawfish SV calling steps are to: (i) identify clusters of SV-associated alignment signatures from mapped sequencing reads; (ii) assemble/polish SV-associated reads from each cluster into one or more haplotype consensus sequences; (iii) merge putative SV haplotypes across samples; (iv) align haplotypes back to a reference sequence; (v) genotype each SV via assessment of relative read support for each local haplotype; and (vi) further refine SV type classification based on the supporting depth signature for unbalanced SV candidates. Details for each of these SV calling operations are described in Supplementary Methods.

In addition to using local haplotype assembly, all sawfish SVs are modeled and genotyped at the breakpoint level, which allows all breakpoints to be consistently reported at basepair resolution, with any breakpoint insertion and microhomology sequence detected and described in the output. For multi-breakpoint events, such as inversions, sawfish will report both the event and its component breakpoints to retain this breakpoint detail level for all cases. Another feature derived from the local haplotype modeling in sawfish is that local SV phasing can be reported in the output wherever two variant haplotypes overlap in the same sample, helping to provide a more detailed description of complex variation regions.

Due to the high resolution of sawfish’s SV calls, and the tendency for this to result in overlapping SV alleles in complex variation regions, we assess single-sample call accuracy on HG002 against a recent similarly high-resolution benchmark, the draft Genome in a Bottle (GIAB) SV benchmark based on the T2T-HG002-Q100 diploid assembly. This benchmark requires the use of updated assessment workflows to harmonize query and benchmark SV representation in complex variation regions, so we test with two independent assessment tools, Truvari (English *et al.* 2022) and hap-eval (https://github.com/Sentieon/hap-eval), to check for consistent qualitative results from both (see Supplementary Methods). Although this strategy introduces additional assessment complexity, compared to the previous mapping-based GIAB SV benchmark (Zook *et al.* 2020), the newer assembly-based benchmark contains roughly three times as many confident SVs (29167 vs 9646), and spot-checking of trial assessment results labeled as false positives or false negatives from each benchmark set has shown a much lower level of assessment artifact compared to the previous mapping-based benchmark.

## 3 Results

The local haplotype assembly implemented in sawfish leads to improved SV calling accuracy in both single-sample and multi-sample joint-genotyping contexts. Here, we assess SV performance for both contexts compared to two state-of-the-art long-read SV callers, Sniffles2 (Smolka *et al.* 2024) and pbsv (https://github.com/PacificBiosciences/pbsv).

### 3.1 Single-sample accuracy

To benchmark SV performance, we take advantage of recent advances from the Telomere-to-telomere consortium HG002 Q100 project (https://github.com/marbl/hg002), which seeks to create a near perfect diploid genome for HG002 based on long-read assembly. This high-quality diploid genome assembly can be used to generate a comprehensive benchmark by aligning assembly contigs back to a standard reference genome. A draft SV benchmark using this approach has been created by GIAB (draft benchmark V0.019-20241113 based on the T2T-HG002-Q100v1.1 diploid assembly, see Supplementary Methods), which we use to assess single-sample SV caller accuracy. Compared to the previous GIAB SV benchmark (Zook *et al.* 2020), this assembly-based benchmark contains roughly three times as many confident SVs (29167 vs 9646) to more comprehensively assess SVs throughout the genome.

All SV callers are first assessed on recent HiFi whole genome sequence (WGS) data for HG002 at 32-fold coverage to characterize accuracy at typical full WGS depth. We assess accuracy against the GIAB T2T SV benchmark using Truvari (English *et al.* 2022) (see Supplementary Methods), finding that the sawfish F1-score of 0.976 is 1.2% higher than Sniffles2 and 1.4% higher than pbsv, as shown in Supplementary Table S1. Given the recent development of the T2T SV benchmark and its associated assessment methods, we check this result by replicating the assessment with an independent method, hap-eval (https://github.com/Sentieon/hap-eval) (see Supplementary Methods) and find that the observed accuracy trend is consistent across assessment methods. The hap-eval sawfish F1-score is 0.977, which in this case is 3.7% and 4.5% higher than Sniffles2 and pbsv, respectively. After this assessment consistency check, all further analysis is consolidated to use Truvari.

In addition to assessing the final SV calls, we also directly assessed sawfish’s assembled SV haplotype sequences for HG002 at 32-fold coverage, by aligning these haplotypes directly to the HG002 Q100 v1.1 diploid assembly (see Supplementary Results). We find that 99.4% of these haplotype sequences align directly to the assembly as expected, consistent with the high precision observed in variant-based accuracy assessments (Supplementary Table S1).

**Figure 1.**
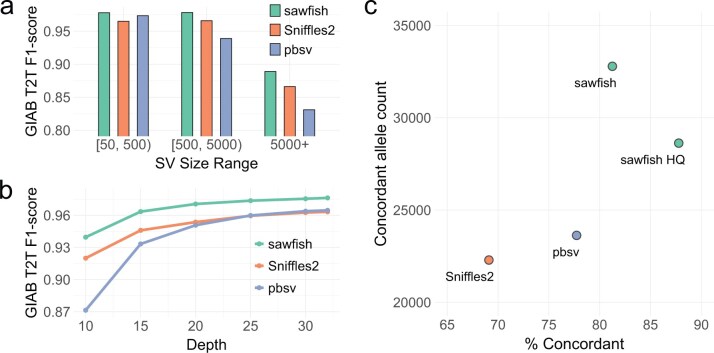
SV caller accuracy assessment. (a) Assessment of SV caller performance on the HG002 GIAB draft T2T assembly-based benchmark for HiFi WGS data from HG002 at 32-fold coverage. Results are stratified by SV size, showing consistently improved F1-score for sawfish across a range of SV sizes. (b) HG002 SV caller performance assessed as in part (a) but for all SV sizes with depth levels subsampled down to 10-fold coverage, showing improved F1-score for sawfish at all coverage levels. (c) Assessment of SV caller joint-genotyping on samples from CEPH Pedigree 1463 generations 2 and 3. For each SV caller, the number of SVs where genotypes are concordant with the pedigree inheritance pattern across all samples are shown compared to the percentage of concordant SVs. “sawfish HQ” shows sawfish results filtered for genotype quality (GQ) ≥40 in all samples. The results show that sawfish calls thousands more concordant SVs than other SV callers while at the same time calling proportionally more concordant SVs, where the proportion of concordant SVs can be increased to over 87% with modest quality filtration that maintains a very high concordant SV count.

We next stratify the accuracy benchmark by SV length into three size ranges: 50 to 499 bases, 500 to 4999 bases, and 5000 bases or more (Fig. 1a, Supplementary Table S2). We observe that sawfish has the highest F1-score in every size range, with a modest F1-score increase of 0.5% for the smallest (50–499 bp) SVs compared to the next-best method, pbsv, but with a growing lead as SV size increases. For SVs of at least 5000 bases, sawfish’s F1-score is over 2.6% and 7.0% higher than Sniffles2 and pbsv, respectively. Thus, we observe that the higher accuracy of sawfish calls extends across SV size ranges. When the benchmark SVs are further separated into insertions and deletions, in addition to size ranges, we continue to see sawfish retain the highest F1-score among the tested SV callers in every SV type category (Supplementary Table S2).

We additionally evaluate how read coverage influences SV call accuracy by running all SV callers on WGS data subsampled at various levels down to 10-fold coverage (Fig. 1b, Supplementary Table S3). We have already shown that sawfish has the highest accuracy at full coverage, and here we observe that sawfish outperforms other methods by even greater margins at lower coverage. At 10-fold coverage the sawfish F1-score is over 2.1% higher than Sniffles2 and 7.9% higher than pbsv. In fact, at 15-fold coverage sawfish achieves roughly the same accuracy as other SV callers at 30-fold coverage, suggesting that population studies could use lower depth without loss of SV accuracy through improved calling and genotyping methods.

To better put the coverage analysis in context, we additionally call SVs at each coverage level via a global assembly process using hifiasm (Cheng *et al.* 2021) and the Phased Assembly Variant (PAV) caller (Ebert *et al.* 2021) (see Supplementary Methods), finding that sawfish can outperform the global assembly approach at 10-fold coverage (Supplementary Fig. S2). Although at higher coverages global assembly shows greater accuracy than any mapping-based SV caller, we observe that sawfish recovers more than half of the F1-score gap between global assembly and either pbsv or Sniffles2 (Supplementary Table S3).

#### 3.1.1 Medically relevant SV benchmark

We compare SV caller performance on the GIAB challenging medically relevant genes benchmark set of 217 SVs (Wagner *et al.* 2022). Although all long-read SV callers are relatively accurate in this context, we observe even higher accuracy for sawfish calls compared to other callers, with sawfish calls including only two false negatives and one false positive, resulting in an F1-score of 0.993 (Supplementary Table S4). This compares to an F1-score of 0.964 for Sniffles and 0.971 for pbsv. This result demonstrates that sawfish methods improvements are not just applicable to comprehensive SV benchmark performance, but SVs in medically relevant genes as well.

### 3.2 Genotype concordance in a large pedigree

While the sawfish haplotype modeling approach has been shown to benefit SV accuracy in individual samples, it also improves joint-genotyping accuracy across multiple samples via merging observations and genotyping at the SV haplotype level. By directly comparing SV haplotypes between samples in the merging process, the method reduces alignment-dependent artifacts introduced in the direct comparison of variants.

To assess joint-genotyping accuracy, we evaluate SVs from HiFi WGS data recently generated for all members of CEPH Pedigree 1463 (Porubsky *et al.* 2024). We jointly genotype SVs across seven samples from the second and third pedigree generations. For each SV, we then evaluate whether the genotypes called across all seven samples are consistent with the known pedigree haplotype inheritance pattern in that region, as described in Kronenberg *et al.* (2024). We summarize the results of this analysis in Fig. 1c and Supplementary Table S5, which show that sawfish not only has 9160 more concordant SVs than either of the other SV callers but also has proportionally more concordant calls at 81.2%.

To evaluate sawfish’s genotype quality score for this application, we filter sawfish calls for a genotype quality of at least 40 in all samples, creating a call set labeled “sawfish HQ” (Fig. 1c, Supplementary Table S5). The sawfish HQ call set still retains 4998 more concordant SVs than the next best caller and also has 87.8% concordance, a substantial gain compared to the next best method, pbsv, with 77.7% concordance. Sniffles2 also provides genotype quality values, so we filter the Sniffles2 calls to require a genotype quality of at least 10 for all samples to get a similar concordance (85.2%), but this requires filtering out 62.6% of the original Sniffles2 concordant SV call set, leaving only 8348 concordant SVs (Supplementary Table S5). This result shows that sawfish’s quality model provides a more effective joint-genotyping solution to achieve both high sensitivity and genotype accuracy.

## 4 Conclusion

Our assessment of long-read SV calling accuracy shows that sawfish’s approach to SV haplotype modeling consistently improves SV accuracy in both single-sample and multi-sample joint-genotyping contexts, and further demonstrates that this accuracy gain is consistent over SV size ranges and across typical WGS coverage levels. In a joint-genotyping context, sawfish calls many more concordant SVs than other callers, while providing a higher enrichment for concordance among all calls. In addition to the advantages of SV haplotype modeling, sawfish’s high accuracy shows the advantages of supporting depth evaluation for large deletions and duplications, native phasing for proximal or overlapping SVs, and an effective genotype quality model which allows for flexible filtration options. These methods will continue to be developed and refined against newly available high-quality assembly-based SV benchmark sets to help realize the full potential of SV discovery through comprehensive long-read genome sequencing.

## Supplementary Material

btaf136_Supplementary_Data

## Data Availability

Sawfish source code, pre-compiled Linux binaries, and documentation are released on GitHub: https://github.com/PacificBiosciences/sawfish. SV calls and assessment results are provided as a Zenodo dataset at https://doi.org/10.5281/zenodo.14898462. All sequencing data and benchmark sets underlying the SV analysis are publicly available as detailed in Supplementary Methods.
