# Supplementary material for: Sawfish: improving long-read structural variant discovery and genotyping with local haplotype modeling
Source: Bioinformatics. 2025 Apr 9;41(4):btaf136. doi: 10.1093/bioinformatics/btaf136 (PMC12000528; doi:10.1093/bioinformatics/btaf136)
Supplement: btaf136_Supplementary_Data [file btaf136_supplementary_data.pdf]

Supplementary Material / Sawfish: Improving long-read structural variant discovery and genotyping with local haplotype modeling

Christopher T. Saunders[1*], James M. Holt[1], Daniel N. Baker[1], Juniper A. Lake[1], Jonathan R. Belyeu[1], Zev Kronenberg[1], William J. Rowell[1], Michael A. Eberle[1]

1: PacBio, Computational Biology, 1305 O'Brien Drive, Menlo Park, CA 94025
*: Corresponding author (csaunders@pacificbiosciences.com)

Supplementary Methods

Single sample SV accuracy assessment

Sequencing data processing

We used publicly available HiFi WGS data for HG002, sequenced to ~32-fold coverage on the Revio system. The unmapped sequencing data can be downloaded from:

https://downloads.pacbcloud.com/public/revio/2022Q4/HG002-rep1/m84011_220902_175841_s1.hifi_reads.bam

The sequencing data is mapped to GRCh38 using pbmm2 version 1.13.1 with the 'CCS' preset option. The mapped reads were subsampled to each test coverage level shown in Figure 1a using the subsample option in samtools[1] version 1.17 based on mapped coverage assessment from mosdepth[2] version 0.2.6.

SV calling from mapped reads

The two SV callers, Sniffles2[3] and pbsv[4], used for comparison in this study were selected based on the following criteria for our single and multi-sample accuracy assessment: (1) support for SV-calling from long reads; (2) SV genotyping; (3) prediction of all common SV types, including deletions, insertions, duplications, breakpoints and inversions; and (4) direct facility to joint-genotype SVs over multiple samples.

SV calls were generated from sawfish version 0.12.9, Sniffles2 version 2.3.3, and pbsv version 2.9.0. The command-line templates for single-sample analysis of each SV caller are as follows:

Program 1 | Command-line template for sawfish single-sample calling.

```
sawfish discover \
  --threads {thread_count} \
  --ref {reference_fasta} \
  --bam {sample_bam}

sawfish joint-call \
  --threads {thread_count} \
  --sample sawfish_discover_output
```

Program 2 | Command-line template for Sniffles2 single-sample calling.

```
sniffles \
  --threads {thread_count} \
  --input {sample_bam} \
  --reference {reference_fasta} \
  --vcf {output_vcf_gz} \
  --tandem-repeats {tandem_repeats_bed}

# Change a few VCF details to allow downstream bcftools usage:
gzip -dc {output_vcf_gz} |\
sed s/None/0/g |\
awk 'BEGIN {OFS = "\t"} {if(/^#CHROM/) $10="HG002"; print}' |\
bcftools view -Oz -o {fixed_output_vcf_gz} -
bcftools index -t {fixed_output_vcf_gz}
```

Program 3 | Command-line template for pbsv single-sample calling.

```
pbsv discover \
  --hifi \
  --tandem-repeats {tandem_repeats_bed} \
  {sample_bam} \
  {sample_svsig}

pbsv call \
  --threads {thread_count} \
  --hifi \
  --types INS,DEL \
  {reference_fasta} \
  {sample_svsig} \
  {output_vcf}
```

SV calling from global assembly

To better understand the performance of sawfish and other mapping-based SV callers, we also compare performance to an SV-calling process based on global assembly, followed by global assembly contig alignment to GRCh38, with SVs called directly from these contig alignments.

To facilitate comparison to mapping-based SV callers, the global assembly pipeline starts with the sequencing data mapped to GRCh38 with titrated depth levels down to 10-fold coverage, so that both mapping and global assembly approaches use the same set of sequencing data at each coverage level. For the global assembly pipeline, the alignment file for each coverage level first has mapping information removed using the samtools `reset` command. Next, the reads are input to the HiFi-human-assembly-WDL pipeline version 1.0.2 installed from:

https://github.com/PacificBiosciences/HiFi-human-assembly-WDL

This pipeline uses hifiasm[5] version 0.20.0 for contig assembly. Next, we use PAV[6] version 2.4.6 to map the assembly contigs to GRCh38 and call variants. PAV calls both small and large variants, so to make the assessment of PAV results comparable to the mapping-based SV output, all variants smaller than 35 bases were filtered out.

Assessment of SV calls against the GIAB draft SV benchmark

Comprehensive assessment of SV calls on HG002 was conducted against the GIAB draft SV benchmark (V0.019-20241113) based on the T2T-HG002-Q100v1.1 diploid assembly aligned to GRCh38. The benchmark variants and confidence regions were downloaded from:

https://ftp-trace.ncbi.nlm.nih.gov/ReferenceSamples/giab/data/AshkenazimTrio/analysis/NIST_HG002_DraftBenchmark_defrabbV0.019-20241113/

This benchmark was modified to exclude sex chromosomes by removing chromosomes X and Y from the confidence regions and additionally to remove small variants by filtering out VCF records without "`INFO/SVTYPE`" values.

Calls were assessed against this benchmark using Truvari[7] version 4.2.2. The Truvari refine procedure with MAFFT[8] alignment was used to harmonize query and truth VCF variant representations. The command-line template for Truvari assessment is provided below.

Program 4 | Command-line template for Truvari assessment against GIAB draft T2T SV benchmark

```
truvari bench \
  --reference {reference_fasta} \
  --includebed {benchmark_region_bed} \
  --base {benchmark_vcf} \
  --comp {input_sv_call_vcf} \
  --output {output_dir} \
  --passonly \
  --pick ac \
  --dup-to-ins

truvari refine \
    --reference {reference_fasta} \
    --regions {output_dir}/candidate.refine.bed \
    --recount \
    --use-region-coords \
    --use-original-vcfs \
    --align mafft \
    {output_dir}

# Produce final assessment summary from combined bench/refine result:
truvari ga4gh \
    --input {output_dir} \
    --output {output_dir}/combined_result \
    --with-refine
```

The Truvari refinement procedure described in this command-line template relies on phased variant representations from all SV callers. Sawfish already natively provides phased output for very close and overlapping SVs, so to apply this workflow to the additional SV callers we used HiPhase[9] version 1.4.1 to phase each SV VCF with respect to the sample read alignment file as follows:

Program 5 | Command-line template for Hiphase-based SV call phasing

```
hiphase \
  --bam {sample_bam} \
  --reference {reference_fasta} \
  --vcf {unphased_sv_vcf} \
  --output-vcf {phased_sv_vcf}
```

Assessment methods for the GIAB draft T2T SV benchmark are still being developed and optimized. To help check the robustness of our Truvari assessment results, we evaluated all SV calls against this SV benchmark using an additional assessment method, hap-eval[10], which provides both an independent assessment and uses a technique that does not rely on local SV

phasing. For the hap-eval performance described in Results and Table S1, we use hap-eval installed from git SHA “e3e2c55” and run the command line below for each SV caller’s output:

Program 6 | Command-line template for hap-eval assessment against GIAB draft T2T SV benchmark

```
hap_eval \
  --reference {reference_fasta} \
  --interval {benchmark_region_bed} \
  --base {benchmark_vcf} \
  --comp {input_sv_call_vcf}
```

Assessment of SV calls against the GIAB CMRG SV benchmark

The GIAB CMRG SV Benchmark 1.0 variant and confidence regions were downloaded from:

https://ftp-trace.ncbi.nlm.nih.gov/ReferenceSamples/giab/release/AshkenazimTrio/HG002_NA24385_son/CMRG_v1.00/GRCh38/StructuralVariant

SV calls were assessed against this benchmark using Truvari version 4.2.2, but with a simplified analysis not requiring the Truvari “refine” method, as described in the command-line template below.

Program 7 | Command-line template for Truvari assessment against GIAB CMRG SV benchmark

```
truvari bench \
  --reference {reference_fasta} \
  --includebed {benchmark_region_bed} \
  --base {benchmark_vcf} \
  --comp {input_sv_call_vcf} \
  --output {output_dir} \
  --passonly \
  --pick ac \
  --dup-to-ins
```

SV context annotation

From the Truvari assessment of sawfish SV call accuracy against the HG002 T2T benchmark set, we further analyzed subsets of sawfish calls by genomic context. To annotate SV calls by context we acquired GRCh38 region tracks in BED format annotating mobile elements (Alu and LINE), tandem repeats (subdivided into STR and VNTR regions according to motif size) and segmental duplications. Mobile element tracks were created by extracting the ‘Alu’ and ‘LINE’ regions from the UCSC genome browser RepeatMasker table:

http://hgdownload.cse.ucsc.edu/goldenPath/hg38/database/rmsk.txt.gz

Tandem repeat tracks were obtained from:

http://hgdownload.cse.ucsc.edu/goldenPath/hg38/database/simpleRepeat.txt.gz

…and divided by tandem repeat motif size of 7 or greater ('VNTR'), and the remaining smaller motifs ('STR').

Finally, segmental duplications annotations were extracted from:

http://hgdownload.cse.ucsc.edu/goldenPath/hg38/database/genomicSuperDups.txt.gz

SV call sets in VCF format were annotated and counted by intersecting the variant file with each annotation track using bedtools[11] as follows:

Program 8 | Command-line template for SV annotation track count

```
bedtools intersect -header -wa -u -a {sv_call_vcf} -b {annotation_bed_file}
```

To understand the contexts of different sets of SV calls, we performed a simple enrichment analysis comparing the context counts of each SV call set of interest to the context counts of all SVs in the HG002 T2T SV benchmark set. The enrichment ratio for each context is the fraction of SVs with the given context among all SVs in the call set of interest, divided by the fraction of SVs with the given context among all SVs in the benchmark set.

Joint genotyping accuracy assessment

Sequencing data processing

We obtained HiFi whole genome sequencing data for seven samples from the 2nd and 3rd generation of CEPH Pedigree 1463. All data were generated as part of the recent Porubsky et al. study[12]. The samples are NA12877, NA12878, NA12879, NA12881, NA12882, NA12885, and NA12886. All sample data can be accessed from the platinum pedigree AWS open data registry at https://registry.opendata.aws/platinum-pedigree/. For each sample, all reads were mapped to the GRCh38 reference with pbbm2 version 1.10.0 using the "HIFI" preset option.

SV calling

The same SV caller versions used for single-sample calling on HG002 are applied for joint-genotyping on the Platinum Pedigree samples. The command-line options for joint-genotyping on all SV callers are the same as those used for joint-genotyping, with the exception that all samples from CEPH pedigree generations 2 and 3 are included as input to the joint genotyping step using each SV callers sample input format (discovery directory, snf file or svsig file for sawfish, Sniffles2 and pbsv, respectively).

Prior to assessment of joint genotyping concordance, SV joint-genotyping output from all 3 callers are processed in the same way to restrict the evaluated SV set to passing SVs at least 50 bases in length with all breakend records filtered out.

Assessment of SV call concordance on the Platinum Pedigree

SV genotype concordance with the Platinum Pedigree is assessed using the pedigree genotype concordance methods described in Kronenberg et al.[13] . For each SV call, this concordance evaluation assesses if the genotypes in all samples are concordant with the known pedigree haplotype inheritance pattern for the given region. The "`concordance`" software used to make this assessment can be compiled and run according to the documentation here:

https://github.com/Platinum-Pedigree-Consortium/Platinum-Pedigree-Inheritance/blob/main/analyses/Concordance.md

The specific software version used is from git SHA "1e3516f". Among the evaluated SV callers, we compare both the total count of concordant SV calls and the percentage of all concordant and discordant calls which are assessed as concordant.

Sawfish SV calling methods

Candidate discovery

The first 'discover' step of sawfish is run once on each input sample. In this step each sample is scanned for SV evidence, which are clustered and used to assemble a set of candidate SV alleles. Details of this step are provided below.

Scanning sample alignments

The method scans over all mapped reads in the genome to identify clusters of breakpoint-associated read signatures and to create regional depth bins. Reads are filtered from this scan if they are flagged as unmapped, secondary, QC failed or duplicate. Reads with gap compressed identity less than 0.97 are filtered out as well. Reads with mapping quality less than 10 are disqualified from breakpoint evidence scanning but still used to find regional sequencing depth.

Breakpoint evidence is gathered from indel and split read annotations in the read alignments. In each case a simple breakpoint candidate is created with breakends matching the location and orientation implied by the corresponding read alignment feature. Note that split read evidence is parsed from primary alignments only. Additional unpaired breakend evidence is gathered from soft-clipped read edges. Such unpaired breakends are used to assemble large insertions when candidate breakend pairs are found in the expected orientation (details below). Soft-clipped read ends only contribute to breakend candidates when at least 500 bases of the read are soft-clipped on one end, and no clipping is found on the other end of the read.

The average depth of each 2kb bin across the genome is also found while scanning alignments for breakpoint evidence. Gaps created by splitting the read into primary and supplementary alignments are accounted for in the depth calculation, but not the alignment indels.

Clustering breakpoint evidence

Breakpoint evidence from individual reads is clustered into candidate breakpoint clusters as follows: Each breakpoint evidence observation is treated as a cluster with a supporting read count of 1. Breakpoint clusters with a matching breakend orientation are tested for their total breakpoint distance, defined as the sum of the distance between each of their breakends. If the breakpoint distance between the clusters is 500 bases or less, the breakpoint clusters are merged, such that each merged breakend extends from the minimum of the two breakend start positions, and the maximum of the two breakend end positions. The merged supporting read evidence of a cluster is the sum of the two input clusters. After merging is completed, clusters with only a single supporting read are discarded as noise. All others comprise the candidate breakpoint cluster set.

Candidate breakpoint cluster refinement

In the breakpoint cluster refinement process, candidate breakpoints are assembled into SV haplotype contigs, which are then aligned back to the genome to generate candidate SVs used in downstream merging and genotyping steps.

Refinement begins by defining the regions of the genome used to extract reads for SV contig assembly for each breakpoint candidate. For breakpoint candidates with distant breakends, these will simply be the two breakend regions. For breakpoint candidates that form an indel-like breakend orientation pattern with a breakend distance of 600 bases or less, the assembly region is merged to span the full region between the two breakends. These become single-region candidate breakpoints. Such breakpoints go through an additional clustering step to consolidate all single-region candidate breakpoint regions within 300 bases of each other into a single candidate assembly region, except that the consolidation process is limited to prevent the creation of consolidated regions larger than 8000 bases.

Large insertion candidate generation

Large insertions can only be discovered by the standard breakpoint clustering process if the read mapper represents large insertions in the reported read alignments. Additional candidate large insertions can be identified from local soft-clipped read alignment patterns as follows. When a pair of left-anchored and right-anchored soft-clipped breakend candidates are found such that the left and right breakends are within 500 bases, these are converted into large insertion candidate assembly regions if they aren't already overlapping a candidate assembly region from the standard cluster refinement process.

Consensus contig generation

The first step of contig generation is obtaining the subsequences of the reads around each candidate breakpoint position. To do so, reads mapped near the candidate breakpoint are enumerated with the same filtration used for breakpoint evidence discovery. For each read, a trimmed subread is extracted comprising 300 bases of read sequence in each direction from the last base mapped on each side of the candidate breakpoint location. These trimmed reads are additionally filtered out if they lack breakpoint evidence, requiring either an indel at least 25 bases long, or a soft-clipped segment within the trimmed region. Note for the special case of large insertion candidates discovered from paired breakend clusters, no read trimming is applied

on the soft-clipped side of the candidate breakend, because the length of the large insertion isn't known for this case. If there are more than 100 trimmed reads for a given breakpoint candidate, the reads are deterministically subsampled to 100.

The trimmed reads are next clustered and polished into a set of contigs using a simple iterative procedure to assign each read to a partial order alignment (POA) graph using the spoa library[14]. Each of these graphs are generated from previously evaluated trimmed reads, and each is interpreted as being sampled from the same underlying haplotype combined with sequencing noise. Each trimmed read is successively aligned to each POA graph using a linear gap alignment with weights of 1, -3, -1 for match, mismatch, and gap, using wavefront alignment[15]. If at least one read alignment has an aligned read length of 100 or higher, and an alignment score normalized by the aligned read length of 0.96 or higher, then the trimmed read is assigned to the POA graph with the highest normalized alignment score, and the POA graph is updated to include the new trimmed read. If the read does not have a sufficiently high-quality alignment to any existing POA graph, then the read is used to start a new POA graph so long as this would not create more than 8 graphs, otherwise the read is filtered out of the consensus contig generation procedure. After all trimmed reads have been processed, the POA graphs are filtered to remove cases with less than 2 supporting reads, and the top P remaining POA graph clusters by supporting read count are used to generate a consensus contig for downstream steps, where P is the local ploidy count.

Candidate SV generation

Top contigs generated for each breakpoint cluster are processed for candidate SV information by locally aligning these back to the expected reference locations. For indel-like contigs assembled from reads over a single region of the reference, the contigs can simply be aligned back to a similar segment of the reference sequence and all indels over the minimum size (35 by default) extracted from the alignment as SV candidates.

For all other types of contigs, a synthetic derived chromosome reference is created by appending the two reference region segments corresponding to each candidate breakend location, possibly with one of the reference segments reverse-complemented according to the candidate breakend orientation pattern. In this way the contig can be aligned to the synthetic reference with an apparent large deletion extending between the two reference regions, and this alignment can be processed back into a pair of breakends of any orientation, which generalizes to handle all types of SV breakpoints.

Whether using the small indel or generalized breakpoint reference alignment procedure, each breakpoint alignment can be standardized by left-shifting its position and finding the full breakend homology range and insertion sequence.

Joint calling

The second 'joint-call' step of the sawfish pipeline enables candidate SVs to be analyzed across multiple samples. The primary steps are: (1) consolidation of SV candidates which are duplicated across samples; (2) evaluation of read support for the deduplicated candidate SV haplotypes to

genotype the SV in each sample; (3) evaluation of depth support for large copy-changing events; and (4) reporting all SVs as a VCF jointly genotyped across all samples.

Duplicate haplotype consolidation

As a first step to duplicate haplotype merging, overlapping haplotypes from all samples are pooled into groups from which duplicates are found and consolidated. This pooling procedure is run separately within the set of indel-like SV candidates consolidated to a single reference region (as described in the candidate discovery section above), and all other candidates associated with multiple reference regions. For indel-like candidates, the candidate pool is found from all intersecting candidate regions. For all other SV candidates associated with two reference regions, pools are composed of candidates where both reference regions are within 100 bases of at least one other candidate in the pool.

Within each candidate haplotype pool, candidates are clustered into duplicate groups based on pairwise testing first for matching breakpoints, then for very high haplotype sequence similarity if the breakpoints aren't an exact match, with an exception made to exclude duplicate merging of haplotypes candidates from the same sample. Haplotype sequence similarity is determined by using a linear gap aligner with 1, -3, -2 for match, mismatch, and gap scores. If the resulting alignment score normalized by the aligned haplotype length is at least 0.97 then the haplotypes are treated as duplicates. Within each duplicate haplotype group, one member is chosen as representative based on having the highest supporting read count from contig assembly, or longest contig size when supporting read count is tied.

Genotyping

SVs are genotyped in the context of the overlapping haplotype pools created for the purpose of duplicate haplotype identification. Each breakend of each SV allele is evaluated by aligning segments of locally mapped reads to the corresponding segment of the SV haplotype assembly, in addition to aligning these reads to the reference sequence and other SV alleles in the overlapping haplotype pool. The segments of the read, reference and SV haplotypes selected for this purpose on each candidate SV breakend are extended up to 500 bases from the non-anchored edge of the breakend's homology range. Alignment is scored using a linear gap aligner with 1, -3, -2 for match, mismatch, and gap scores, with alignment scores normalized by the aligned read segment length.

The alignment scores for each read are next converted into support counts, where each read could be identified as uniquely consistent with either the haplotype of the SV allele in question, the reference haplotype or a candidate overlapping SV haplotype. Candidate overlapping haplotypes are only identified for local indel-like SV candidates. The overlapping case can be from a second SV haplotype assembled from reads in the given sample. If a second haplotype wasn't assembled for the sample, then overlapping SV haplotypes from other samples (among those remaining after merging duplicate haplotypes) are compared to find the overlapping SV haplotype with the highest support from all sample reads. If such an overlapping case is found it is added as a second 'guest' candidate haplotype.

Each read's support for the SV haplotype is evaluated separately for each breakend of a given breakpoint. A read is counted as supporting an SV allele if its alignment score to the SV haplotype is better than the reference haplotype for any breakend. A read can only support the reference haplotype if the reference haplotype alignment has a higher score on all evaluated breakends. This arrangement helps to counteract various forms of reference bias in the alignment scores. Reads supporting an overlapping SV haplotype are internally recorded for the overlapping haplotype but converted into reference allele support for both quality score calculations and in the final VCF allele count output, per the reporting convention used by most available SV calling tools.

Quality model

The sawfish quality scores are generated from a simplified model which generates qualities directly from the read support counts for each allele. Ideally the scores of read alignments to each allele haplotype would be used instead of counts, but thus far the count approximation hasn't been attributed to any substantial fraction of SV genotyping errors.

For each SV allele and each sample, we solve for the posterior probability of diploid genotypes, *G*, given the observed read counts at the SV loci, *D* as follows

$$P(G|D) \propto P(D|G)P(G)$$

The diploid genotypes are *G* = {*ref, het, hom*} representing 0,1 or 2 copies of the SV allele. The genotype prior is

$$P(G) = \begin{cases} \theta & \text{if} \quad het \\ \theta/2 & \text{if} \quad hom \\ 1 - \theta 3/2 & \text{if} \quad ref \end{cases}$$

where $\theta = 5 \times 10^{-4}$.

The genotype likelihood is found as follows

$$P(D|G) = \prod_{d \in D} P(d|G)$$

treating each read observation $d \in D$ as independent. The read likelihood is

$$P(d|G) = \sum_{a \in A} P(d|a)P(a|G)$$

where *A* = {*ref, alt*} are the SV alleles in the model representing the reference and SV haplotypes. As previously discussed, support for any overlapping SV haplotypes is counted towards the reference allele for the purpose of the genotype quality model, which is intentionally simplified to represent only one SV haplotype at a time.

Considering the terms in the read likelihood, the allele likelihood *P(d|a)* is set from the read allele support counts using a single erroneous read support count probability $e = 1 \times 10^{-5}$ for all

cases. The allele probabilities *P(a|G)* are the simple allele fractions (0, 0.5, 1) associated with each genotype.

Evaluating read coverage support for larger SV calls

After the above described read-based genotyping steps, all deletion and duplication candidates of at least 50kb are additionally evaluated for an SV depth signature consistent with the SV type. If the supporting SV depth signature is not found, the corresponding SV breakpoint is still reported in the VCF output, but as a set of breakend (BND) records, rather than as a deletion or duplication. In this way, breakpoints comprising larger multi-breakpoint complex SVs can still be reported in the VCF output without compromising the precision of simpler copy-number changing deletion and tandem duplication events.

The criteria for a consistent depth signature in each sample is based on the average depth of the interior region of the SV is compared to the average depth in the 6kb regions to the left and right flank. The ratio of the interior to flank depth must be no more than 0.8 for a deletion and no less than 1.2 for a duplication. In a multi-sample context, a consistent depth signature is required in at least half of samples with a non-reference genotype to retain the SV type as a deletion or duplication. While this approach applies a very simple heuristic to assess SV support for each large SV, this has been found to be effective in filtering typical complex SV breakpoints from true large deletion and duplication signatures.

Supplementary Results

Data availability

We have uploaded the SV caller VCF files and assessment results, together with templates for our assessment scripts, to the following Zenodo dataset:

https://doi.org/10.5281/zenodo.14898462

SV context analysis

We sought to further characterize sawfish's performance on HG002 by analyzing SV context. To do so we first characterize all SVs in the HG002 T2T benchmark set as associated with an Alu or LINE mobile element sequence, an STR or VNTR track or a segmental duplication (see Supplementary Methods). We then characterize two other SV call sets related to sawfish HG002 performance: 1) all true positive SVs called by sawfish which are missed by both Sniffles2 and pbsv, and 2) all sawfish false negatives. The same SV context analysis applied to the full benchmark is applied to these two benchmark subsets, and the proportion of each represented context is compared to the full benchmark (Table S6). By this analysis we can see that sawfish's calling improvements are generally applicable across all assessed SV contexts. We note that sawfish does seem to perform particularly well on SVs associated with Alu sequences in the reference, these are enriched almost 1.5-fold in the sawfish unique true positives and are

disproportionately reduced (0.75 enrichment) among sawfish false negatives. SVs associated with segmental duplications appear to be substantially enriched in both sawfish unique true positives (2.17) and in false negatives (3.49). This could be interpreted as an indication that SVs in segmental duplications are more difficult to call for all tested SV callers, so these calls are missed at a higher-than-average rate, and when they are called, it is more likely to be by only one SV caller.

We note that for this analysis, the count of all true positive SVs called by sawfish but not the other SV callers is conservative, and a lower-bound on the true count. These SV call sets are extracted from Truvari outputs, and for certain cases Truvari refinement can make it difficult to directly compare the performance of an individual SV call between all three SV callers.

Assessment of SV calling from global assembly

The results of this global assembly assessment are summarized together with the mapping-based SV calling results in Figures S1 and S2, together with Tables S1, S2, S3, and S4. As expected, we observe that sawfish tends to have higher accuracy at lower sequencing depth where prior information from the reference can help to supplement more limited sequencing data. At 32-fold coverage SV calls from global assembly show higher accuracy, however we observe that sawfish already recovers more than half of the F1-score gap between global assembly and either pbsv or Sniffles2. The remaining F1-score improvement of 0.9% over sawfish is still substantial, and while there may be some question of circularity between these variant calls and a benchmark based on a similar global assembly discovery process, a substantial portion of the calling improvements could be useful to suggest further methods development for mapping-based SV calling.

With this in mind, we investigated one SV category where global assembly-based SV calling is particularly advantageous: SVs of 5kb or longer. For these SVs, the F1-score improvement over sawfish is 7.9%; 4.8% for deletions and 10.3% for insertions (Figure S1a and Table S2). Reviewing a random sample of the large insertions called only by global assembly showed many large insertions with extensive breakpoint homology of 500 bases or more, and additional cases where mapping-based methods were able to call tandem duplications, but the global assembly (including the T2T benchmark set) expressed the same variant more precisely as a large insertion. The former case is an example of a variant type that could be addressed by improvements to the mapping-based SV callers, while the latter case appears potentially addressable as an improvement to large SV benchmarking assessment methods. While a more comprehensive analysis of the global assembly vs mapping-based SV call sets is beyond the scope of this study, our assessment suggests this could be a productive path to find further methodological improvements.

Sawfish SV haplotype quality

In addition to assessing the accuracy of sawfish SV calls, we can also use the HG002 T2T diploid assembly to directly evaluate sawfish's assembled SV haplotypes, by aligning these directly to the T2T assembly. This provides a direct measure of the effectiveness of the assembly

process without the additional complications of genotyping and comparisons between SV call sets.

Sawfish always provides a BAM file of aligned SV haplotypes corresponding to its SV call output. This output is used as a starting point for the SV haplotype assessment. These haplotype sequences were converted to fastq and mapped directly to the HG002 diploid assembly. We use the HG002 assembly v1.1 from:

https://s3-us-west-2.amazonaws.com/human-pangenomics/T2T/HG002/assemblies/hg002v1.1.fasta.gz

Conversion to fastq and mapping with minimap2[16] version 2.26.0 are detailed below. The minimap2 version and parameters have been set to replicate pbbm2 v1.13.1 'CCS' presets, but with secondary mapping output.

Program 9 | Command-line template for sawfish SV haplotype contig mapping to HG002 diploid assembly

```
samtools reset {sawfish_haplotype_bam} |\
samtools fastq > {haplotype_fastq}

minimap2 -k 19 -w 19 -O 6.26 -E 2.1 -A 1 -B 4 -z 400.50 -r 2000 -g 5000 -Y
-L --eqx --secondary=yes -a {hg002_assembly_reference} {haplotype_fastq} |\
samtools sort -o {hg002_mapped_haplotype_bam} --write-index
```

All haplotype alignments are classified as either 1) unmapped, 2) split alignments, 3) having a large difference with the assembly (indel or soft-clipped region longer than 20 bases), or 4) aligned as expected. If a secondary alignment was available, only the best alignment for the haplotype was retained. By these criteria, only 0.6% of 33,144 SV haplotypes had unexpected alignments: none were split, 7 were unmapped, and 192 had a large difference with the assembly. The 192 alignments with large differences could be further decomposed into (non-disjoint) sub-types of deletions (109), soft-clipped edges (50) and insertions (45). Examining a random subset of these cases suggested they were highly enriched for alignments to tandem repeat regions and in the flanking edges of the haplotype.

Compute requirements

Sawfish has been designed to prioritize accuracy while still operating on practical compute requirements.

For a typical ~30x HiFi sample analyzed on 16 threads, the sawfish discover step should complete in about 25-40 minutes and the joint-call step should complete in about 5 minutes. Running the joint-call step on 7 samples at 30-100x depth completes in about 1 hour on 16 threads. In general, runtime response to thread count is expected to be nearly linear for both discover and joint-call steps.

The sawfish joint calling scheme has been designed for pedigree-scale analysis and is not expected to provide a practical solution for larger cohorts. For an example of how performance

scales as a function of sample count, sawfish joint calling has completed on 47 HPRC samples in testing, which required 1044 minutes on 64 threads. Future changes to the joint-calling design could make larger cohorts more practical.

Memory requirements for the sawfish discover step are less than 8Gb/thread so long as at least 3-4 threads are selected. The joint-call step should require substantially less memory, it has been tested at scale with a 1Gb/thread allocation and completes all tests within that limit.

For both the HG002 32x single sample analysis and the CEPH-1463 pedigree 7 sample joint-calling analysis, the SV callers used in this study's accuracy assessment were benchmarked for runtime on a controlled compute environment (16 core allocation on an AMD EPYC 7H12 processor). Results are summarized in Table S7. All assessments are provided as wall time given the fixed 16 core allocation, while following the documented best practices for parallelization of each caller. This benchmark is designed to reflect a typical use case on modern hardware but may not be optimal for callers with limited parallelization, such as pbsv. For most cases the 16-core allocation was controlled by a thread count argument, as shown in the command-line templates above for each caller. For the pbsv discover step, such an option is not available, so the analysis is parallelized by chromosome as shown below.

Program 10 | Command-line template for pbsv discover step parallelized by chromosome

```
samtools view -H {sample_bam} |\
grep '^@SQ' |\
cut -f2 |\
cut -d':' -f2 |\
xargs -P {thread_count} -I {} \
bash -c 'pbsv discover --hifi --tandem-repeats {tandem_repeats_bed}
{sample_bam} ${sample_svsig_prefix}.$@.svsig.gz --region $@' _ {}
```

Supplementary Figures

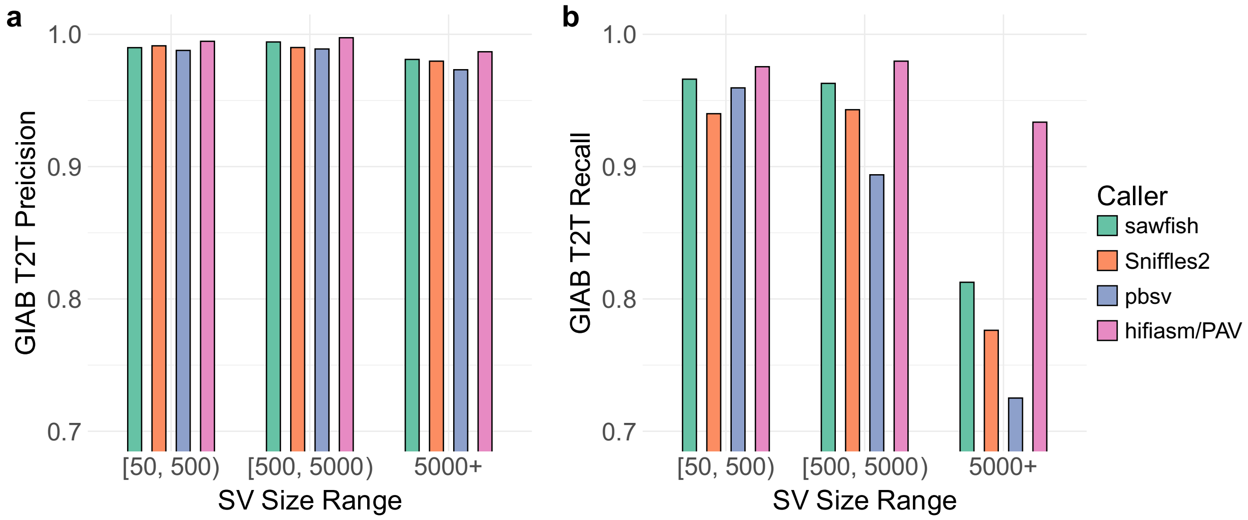

Figure S1 | SV caller accuracy as a function of SV size. *Assessment of all mapping-based SV callers in addition to SV calling results from global assembly (hifiasm/PAV). All SVs are assessed against the GIAB T2T benchmark set and stratified into 3 SV size categories to summarize precision (a) and recall (b).*

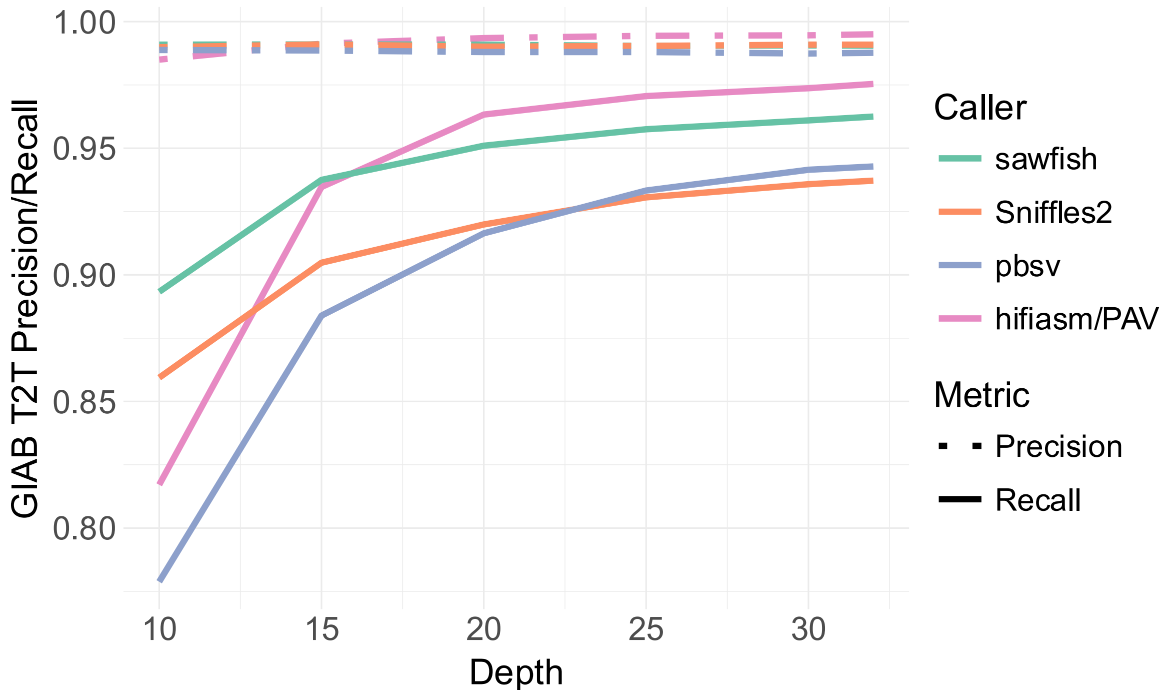

Figure S2 | SV caller accuracy as a function of depth. *Assessment of all mapping-based SV callers in addition to SV calling results from global assembly (hifiasm/PAV). All SVs are assessed against the GIAB T2T benchmark set for aligned read inputs subsampled from 32-fold to 10-fold coverage to summarize precision and recall at each coverage level.*

Supplementary Tables

Table S1 | SV assessment results on GIAB draft T2T SV benchmark using Truvari or hap-eval for 32-fold (full) WGS coverage input.

SV Caller	Assessment Method	F1-Score	Recall	Precision
sawfish	Truvari	0.9763	0.9625	0.9905
Sniffles2	Truvari	0.9633	0.9372	0.9909
pbsv	Truvari	0.9647	0.9428	0.9877
hifiasm/PAV	Truvari	0.9851	0.9754	0.9950
sawfish	hap-eval	0.9768	0.9697	0.9841
Sniffles2	hap-eval	0.9424	0.9221	0.9638
pbsv	hap-eval	0.9351	0.9278	0.9424
hifiasm/PAV	hap-eval	0.9808	0.9716	0.9901

Table S2 | SV assessment results on GIAB draft T2T SV benchmark using Truvari with results binned into 3 SV size ranges, and 3 SV type groups for all SVs (All), deletions (Del), or insertions (Ins).

SV Caller	Size Range	SV Types	F1-Score	Recall	Precision
sawfish	[50, 500)	All	0.9779	0.9661	0.9899
sawfish	[500, 5000)	All	0.9783	0.9629	0.9942
sawfish	5000+	All	0.8889	0.8126	0.9810
Sniffles2	[50, 500)	All	0.9650	0.9400	0.9913
Sniffles2	[500, 5000)	All	0.9659	0.9430	0.9900
Sniffles2	5000+	All	0.8662	0.7763	0.9797
pbsv	[50, 500)	All	0.9734	0.9595	0.9878
pbsv	[500, 5000)	All	0.9390	0.8938	0.9889
pbsv	5000+	All	0.8310	0.7251	0.9732
hifiasm/PAV	[50, 500)	All	0.9850	0.9755	0.9947
hifiasm/PAV	[500, 5000)	All	0.9884	0.9797	0.9974
hifiasm/PAV	5000+	All	0.9595	0.9336	0.9868
sawfish	[50, 500)	Del	0.9736	0.9611	0.9865
sawfish	[500, 5000)	Del	0.9787	0.9743	0.9832
sawfish	5000+	Del	0.9328	0.9040	0.9636
Sniffles2	[50, 500)	Del	0.9595	0.9318	0.9888
Sniffles2	[500, 5000)	Del	0.9650	0.9461	0.9846
Sniffles2	5000+	Del	0.9245	0.8743	0.9809
pbsv	[50, 500)	Del	0.9697	0.9588	0.9808
pbsv	[500, 5000)	Del	0.9569	0.9446	0.9696
pbsv	5000+	Del	0.9318	0.8920	0.9752
hifiasm/PAV	[50, 500)	Del	0.9812	0.9696	0.9931
hifiasm/PAV	[500, 5000)	Del	0.9883	0.9838	0.9928
hifiasm/PAV	5000+	Del	0.9774	0.9719	0.9830
sawfish	[50, 500)	Ins	0.9806	0.9694	0.9922
sawfish	[500, 5000)	Ins	0.9784	0.9595	0.9981
sawfish	5000+	Ins	0.8606	0.7553	1.0000
Sniffles2	[50, 500)	Ins	0.9687	0.9454	0.9931
Sniffles2	[500, 5000)	Ins	0.9665	0.9420	0.9923
Sniffles2	5000+	Ins	0.8250	0.7132	0.9783
pbsv	[50, 500)	Ins	0.9761	0.9599	0.9928
pbsv	[500, 5000)	Ins	0.9334	0.8770	0.9976
pbsv	5000+	Ins	0.7554	0.6182	0.9710
hifiasm/PAV	[50, 500)	Ins	0.9874	0.9793	0.9957
hifiasm/PAV	[500, 5000)	Ins	0.9887	0.9784	0.9992
hifiasm/PAV	5000+	Ins	0.9490	0.9112	0.9901

Table S3 | SV assessment results on GIAB draft T2T SV benchmark using Truvari for 32-fold (full) WGS coverage and subsampled levels down to 10-fold coverage.

SV Caller	Coverage	F1-Score	Recall	Precision
sawfish	10	0.9396	0.8933	0.9909
sawfish	15	0.9635	0.9375	0.9910
sawfish	20	0.9706	0.9510	0.9909
sawfish	25	0.9737	0.9575	0.9904
sawfish	30	0.9755	0.9610	0.9906
sawfish	32	0.9763	0.9625	0.9905
Sniffles2	10	0.9200	0.8594	0.9899
Sniffles2	15	0.9460	0.9048	0.9910
Sniffles2	20	0.9537	0.9199	0.9901
Sniffles2	25	0.9596	0.9306	0.9904
Sniffles2	30	0.9626	0.9358	0.9909
Sniffles2	32	0.9633	0.9372	0.9909
pbsv	10	0.8714	0.7788	0.9888
pbsv	15	0.9333	0.8839	0.9886
pbsv	20	0.9508	0.9164	0.9880
pbsv	25	0.9599	0.9333	0.9880
pbsv	30	0.9639	0.9415	0.9874
pbsv	32	0.9647	0.9428	0.9877
hifiasm/PAV	10	0.8933	0.8171	0.9850
hifiasm/PAV	15	0.9622	0.9347	0.9913
hifiasm/PAV	20	0.9782	0.9633	0.9935
hifiasm/PAV	25	0.9823	0.9706	0.9944
hifiasm/PAV	30	0.9840	0.9737	0.9946
hifiasm/PAV	32	0.9851	0.9754	0.9950

Table S4 | SV assessment results on GIAB Challenging Medically Relevant Genes (CMRG) benchmark. *Assessment results of each SV caller against the GIAB CMRG benchmark using Truvari. FN: False Negative, FP: False Positive.*

SV Caller	F1-Score	Recall	Total FNs	Precision	Total FPs
sawfish	0.9929	0.9907	2	0.9951	1
Sniffles2	0.9640	0.9630	8	0.9650	7
pbsv	0.9714	0.9583	9	0.9849	3
hifiasm/PAV	0.9763	0.9676	7	0.9851	3

Table S5 | Pedigree concordant SV calls on 7 samples from CEPH-1463 generations 2 and 3.

SV Call Set	Total SVs	Concordant SVs	Discordant SVs	% Concordant
sawfish	40355	32789	7566	81.25
Sniffles2	32262	22293	9969	69.10
pbsv	30399	23629	6770	77.73
sawfish HQ (GQ >= 40)	32614	28627	3987	87.78
Sniffles2 HQ (GQ >= 10)	9804	8348	1456	85.15

Table S6 | SV context analysis. *Categorization of HG002 SVs by context, and context enrichment relative to all SVs in the HG002 T2T benchmark. Analysis applied to all sawfish false negatives and all sawfish true positives that were uncalled by both Sniffles2 and pbsv. Context labels are not disjoint.*

SV Call Set	Context	Context Count	Total Count	Context Proportion	Enrichment Relative to Benchmark
T2T Benchmark	ALU	3244	28123	0.115	1.000
	LINE	2718	28123	0.097	1.000
	STR	6961	28123	0.248	1.000
	VNTR	18618	28123	0.662	1.000
	SEGDUP	2212	28123	0.079	1.000
Sawfish false negatives	ALU	75	867	0.087	0.750
	LINE	90	867	0.104	1.074
	STR	226	867	0.261	1.053
	VNTR	545	867	0.629	0.950
	SEGDUP	238	867	0.275	3.490
Sawfish unique true positives	ALU	21	123	0.171	1.480
	LINE	13	123	0.106	1.094
	STR	26	123	0.211	0.854
	VNTR	78	123	0.634	0.958
	SEGDUP	21	123	0.171	2.171

Table S7 | Runtime benchmarks. *Comparison of SV caller runtimes for the HG002 32x and CEPH-1463 Pedigree analyses. All results are shown as wall time for the corresponding caller given 16 cores on an AMD EPYC 7H12 processor.*

Analysis	SV Caller	Discover wall time (mins)	Joint-call wall time (mins)	Total wall time (mins)
HG002 32x	sawfish	26.7	2.5	29.1
	Sniffles2	N/A	N/A	1.5
	pbsv	1.5	137.0	138.5
CEPH-1463 Pedigree (7 samples)	sawfish	314.7	61.4	376.1
	Sniffles2	31.8	0.4	32.1
	pbsv	42.3	619.0	661.3

References

1. Danecek, P. *et al.* Twelve years of SAMtools and BCFtools. *GigaScience* **10**, giab008 (2021).

2. Pedersen, B. S. & Quinlan, A. R. Mosdepth: quick coverage calculation for genomes and exomes. *Bioinformatics* **34**, 867–868 (2017).

3. Smolka, M. *et al.* Detection of mosaic and population-level structural variants with Sniffles2. *Nat. Biotechnol.* 1–10 (2024) doi:10.1038/s41587-023-02024-y.

4. pbsv - PacBio structural variant (SV) calling and analysis tools. https://github.com/PacificBiosciences/pbsv.

5. Cheng, H., Concepcion, G. T., Feng, X., Zhang, H. & Li, H. Haplotype-resolved de novo assembly using phased assembly graphs with hifiasm. *Nat Methods* **18**, 170–175 (2021).

6. Ebert, P. *et al.* Haplotype-resolved diverse human genomes and integrated analysis of structural variation. *Science* **372**, (2021).

7. English, A. C., Menon, V. K., Gibbs, R. A., Metcalf, G. A. & Sedlazeck, F. J. Truvari: refined structural variant comparison preserves allelic diversity. *Genome Biol.* **23**, 271 (2022).

8. Katoh, K. & Toh, H. Recent developments in the MAFFT multiple sequence alignment program. *Brief. Bioinform.* **9**, 286–298 (2008).

9. Holt, J. M. *et al.* HiPhase: jointly phasing small, structural, and tandem repeat variants from HiFi sequencing. *Bioinformatics* **40**, btae042 (2024).

10. hap-eval: A VCF comparison engine for structural variant benchmarking. https://github.com/Sentieon/hap-eval.

11. Quinlan, A. R. & Hall, I. M. BEDTools: a flexible suite of utilities for comparing genomic features. *Bioinformatics* **26**, 841–842 (2010).

12. Porubsky, D. *et al.* A familial, telomere-to-telomere reference for human de novo mutation and recombination from a four-generation pedigree. *bioRxiv* 2024.08.05.606142 (2024) doi:10.1101/2024.08.05.606142.

13. Kronenberg, Z. *et al.* The Platinum Pedigree: A long-read benchmark for genetic variants. *bioRxiv* 2024.10.02.616333 (2024) doi:10.1101/2024.10.02.616333.

14. Vaser, R., Sović, I., Nagarajan, N. & Šikić, M. Fast and accurate de novo genome assembly from long uncorrected reads. *Genome Res* **27**, 737–746 (2017).

15. Marco-Sola, S., Moure, J. C., Moreto, M. & Espinosa, A. Fast gap-affine pairwise alignment using the wavefront algorithm. *Bioinformatics* **37**, 456–463 (2020).

16. Li, H. Minimap2: pairwise alignment for nucleotide sequences. *Bioinformatics* **34**, 3094–3100 (2018).
